# Advanced Diagnostic and Therapeutic Endoscopy for Early Gastric Cancer

**DOI:** 10.3390/cancers16051039

**Published:** 2024-03-03

**Authors:** Mitsuhiro Fujishiro

**Affiliations:** Department of Gastroenterology, Graduate School of Medicine, The University of Tokyo, 7-3-1, Hongo, Bunkyo-ku, Tokyo 113-8655, Japan; mtfujish@gmail.com; Tel.: +81-3-3815-5411; Fax: +81-3-5800-8799

**Keywords:** early gastric cancer, gastric cancer screening, endoscopic diagnosis, endoscopic mucosal resection, endoscopic submucosal dissection

## Abstract

**Simple Summary:**

Advances in gastric cancer screening and endoscopic technology have made it possible to detect and treat gastric cancer at an early stage. As a result, about 40% of all gastric cancers have now been resected via endoscopic treatment in Japan. In endoscopic diagnosis, magnifying endoscopic observation using band-limited light such as narrow-band imaging (NBI) has significantly improved the accuracy of gastric cancer diagnosis. At the same time, in endoscopic treatment, endoscopic submucosal dissection (ESD) has contributed to notably increasing the number of gastric cancers that can be resected endoscopically. In recent years, the construction of algorithms for endoscopic diagnosis and treatment with a view to personalized medicine has been explored, and the use of endoscopy for gastric cancer management is expected to develop further in the future.

**Abstract:**

Endoscopy is mandatory to detect early gastric cancer (EGC). When considering the cost-effectiveness of the endoscopic screening of EGC, risk stratification by combining serum pepsinogen values and anti-*H. pylori* IgG antibody values is very promising. After the detection of suspicious lesions of EGC, a detailed observation using magnifying endoscopy with band-limited light is necessary, which reveals an irregular microsurface and/or an irregular microvascular pattern with demarcation lines in the case of cancerous lesions. Endocytoscopy enables us to make an in vivo histological diagnosis. In terms of the indications for endoscopic resection, the likelihood of lymph node metastasis and technical difficulties in en bloc resection is considered, and they are divided into absolute, expanded, and relative indications. Endoscopic mucosal resection and endoscopic submucosal dissection are the main treatment modalities nowadays. After endoscopic resection, curability is evaluated histologically as endoscopic curability (eCura) A, B, and C (C-1 and C-2). Recent evidence suggests that the outcomes of endoscopic resection for many EGCs are comparable to those of gastrectomy and that endoscopic resection is the gold standard for node-negative early gastric cancers. Personalized medicine is also being developed to overcome the unmet needs in treatments of EGC, for example the further expansion of indications and newer resection techniques, such as full-thickness resection.

## 1. Introduction

Although the number of patients with gastric cancer is expected to decrease in the future due to the decline in the number of people infected with *Helicobacter pylori* (*H. pylori*) and the spread of *H. pylori* eradication therapy, the incidence of gastric cancer still ranks fifth among all cancer types (1,089,103), and it caused the fourth largest number of deaths (768,793) in 2020 in the world [1]. With the spread of esophagogastroduodenoscopy (EGD) in daily practice, the early detection of gastric cancer became possible. Further combined with the birth of endoscopic submucosal dissection (ESD) in the late 1990s [2,3], the era in which early-stage gastric cancer without lymph node metastasis can be cured with endoluminal surgery alone has arrived. This review outlines the current status of the endoscopic diagnosis and treatment of gastric cancer, as well as the future prospects in this field.

## 2. The Role of Endoscopy in Gastric Cancer Detection

Japan is one of the few countries where population-based mass screening of gastric cancer has been carried out for a long time. X-ray examination with barium meal had been the only recommended mass screening method until 2016; however, it has been fraught with various problems, such as a low participation rate (less than 50% of target generations) and radiation exposure. In the 2014 edition of the “Gastric Cancer Screening Guidelines Based on Efficacy Evaluation” published by the National Cancer Center in Japan, endoscopy was recommended for population-based mass screening for the first time, along with X-ray examination, because there is sufficient evidence that it has a mortality reduction effect [4,5].

According to the Screening Guidelines, endoscopy once every 2–3 years is recommended for individuals over 50 years old who have an increased risk of gastric cancer. However, it is not practical to perform EGD on every individual over the age of 50 in terms of cost-effectiveness and endoscopist manpower. Since the *H. pylori* infection is involved in the development of gastric cancer in more than 95% of cases, and it is known that the risk of developing gastric cancer increases with the progression of gastric mucosa atrophy and intestinal metaplasia due to *H. pylori* infection, stratified screening according to the risk of gastric cancer should be explored.

**Figure 1:**
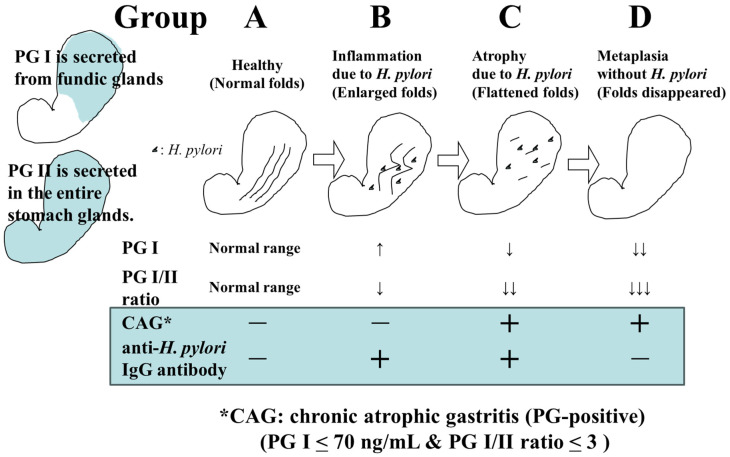
Changes in *H. pylori* infection status, gastric environment, and serum PG and HP levels.

The screening of gastric cancer using the ABC classification is a method that stratifies the risk of developing gastric cancer by combining serum pepsinogen (PG) values, which are serum markers of atrophic gastritis, and anti-*H. pylori* IgG antibody (HP) values [6]. In a previous report, where PGI ≤ 70 ng/mL and PGI/II ratio ≤ 3.0 were defined as PG-positive (with atrophic gastritis), the risk of developing gastric cancer in Group A (HP-negative PG-negative), Group B (HP-positive PG-negative), Group C (HP-positive PG-positive), and Group D (HP-negative PG-positive) was reported to be 0.016%, 0.14%, 0.30%, and 1.1%, respectively [7] (Figure 1). Based on this report, it has been proposed that Group A should not undergo endoscopy (or it should be conducted once every 5 years), Group B should be examined once every 3 years, Group C should be examined once every 2 years, and group D should be examined annually.

However, with the spread of *H. pylori* eradication therapy, there may be more than a few cases of *H. pylori* eradication in Group A; therefore, it is not appropriate to uniformly withhold endoscopy from Group A. It is necessary to take relevant measures such as considering the history of *H. pylori* eradication and performing endoscopy once every 2–3 years for eradication cases. A subgroup analysis has also pointed out that Group B includes patients with a high incidence of gastric cancer (e.g., PGI > 70 ng/mL and PGI/II ratio ≤ 3.0); thus, more optimal risk stratification and appropriate endoscopic intervals should be explored through further examination [8].

When a patient is presumed to be uninfected with *H. pylori*, based on endoscopic findings indicating no atrophy and the existence of regular arrangements of collecting venules (RAC) in the gastric angle [9], the risk of cancer is extremely low and thus endoscopy can be completed in a short time. However, regarding an *H. pylori*-infected or -eradicated patient, when it is determined that the risk of cancer is high based on the endoscopic findings such as the progression of open-type atrophy, the presence of intestinal metaplasia, enlarged folds of the gastric body, and nodular gastritis in the gastric antrum, a more detailed examination is required. The latter two findings are important in classifying patients at high risk of undifferentiated gastric cancer [10,11].

## 3. Endoscopic Diagnosis of Early Gastric Cancer

Elevated and protruded cancers, which are principally characterized as areas with irregularities and a whitish color, are usually picked up by white light observation. When the mucosal surface structure is different from the surroundings, differentiation has to be made as to whether such lesions are non-neoplastic lesions, such as fundus gland polyps, hyperplastic polyps, and intestinal metaplasia, or neoplastic lesions, such as adenomas and cancer. Those with remarkable irregularities, large nodules, and noticeable redness in some parts, and that are >2 cm in size are likely to be cancer.

**Figure 2:**
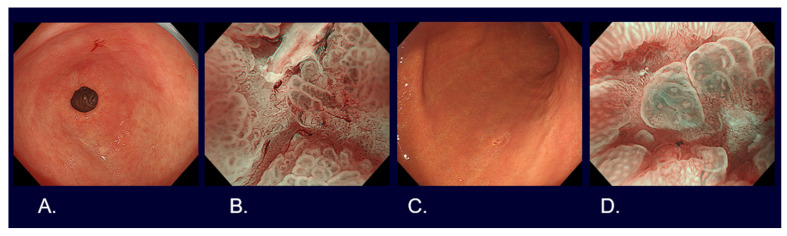
Endoscopic images of early gastric cancers. (**A**) White light observation of type 0-IIc differentiated cT1a without ulcerative findings, <2 cm in size, on the lesser curve of the gastric antrum. (**B**) Narrow-band imaging observation with 80 times magnification of Figure 1A lesion (irregular microvascular pattern with demarcation line). (**C**) White light observation of type 0-IIc undifferentiated cT1a without ulcerative findings, <2 cm in size, on the greater curve of the gastric body. (**D**) Narrow-band imaging observation with 80 times magnification of Figure 1C lesion (irregular microvascular pattern with demarcation line).

Flat and depressed cancers, which are characterized as areas with irregularities (a stellate shape) and a reddish or whitish color, are usually picked up by white light observation. Although it is often difficult to distinguish between cancer and non-cancer, new technologies are helping to overcome such difficulties. Detailed observation using magnifying endoscopy with band-limited light, such as narrow-band imaging (NBI), reveals an irregular microsurface and/or an irregular microvascular pattern with demarcation lines in the case of cancerous lesions (Figure 2). This new algorithm is known as the magnifying endoscopy simple diagnostic algorithm for gastric cancer (MESDA-G) [12], and it is based on the vessels plus surface (VS) classification system [13]. Although the progress of endoscopic diagnostic technology has been remarkable as described above, histological diagnosis with biopsy has to be conducted to confirm whether a lesion is cancer.

## 4. Guidelines for Endoscopic Resection for Early Gastric Cancers [14,15]

### Indications of Endoscopic Resection for Early Gastric Cancer

Two aspects must be confirmed to determine the indication for endoscopic resection: (i) the possibility of lymph node metastasis must be extremely low, and (ii) it must be possible for the lesions to be endoscopically resected in an en bloc fashion.

“Absolute indication” lesions are defined as those where the risk of lymph node metastasis is estimated to be <1%, and long-term results equivalent to surgical gastrectomy are proven. “Expanded indication” lesions are defined as those where the risk of lymph node metastasis is estimated to be <1% but there is little evidence of long-term results. Furthermore, endoscopic resection for early-stage gastric cancer without lymph node metastasis, which would usually be treated with gastrectomy but where surgery may not be recommended due to various clinical circumstances such as advanced age and a substantial underlining disease, is categorized as a “relative indication”.

Specifically, “absolute indication” lesions are divided into EMR/ESD-adapted lesions and ESD-adapted lesions. The former are clinically differentiated intramucosal carcinomas (cT1a) and ≤2 cm, with no ulcerative findings (UL0). The latter are undifferentiated cT1a and ≤2 cm, with UL0; differentiated cT1a and >2 cm, with UL0; or differentiated cT1a and ≤3 cm, with ulcerative findings (UL1). Lesions can be regarded as an “expanded indication” for ESD provided that the “absolute indication” lesions locally recur as differentiated cT1a after initial ESD/EMR with a C-1 grade of endoscopic curability (eCura).

**Figure 3:**
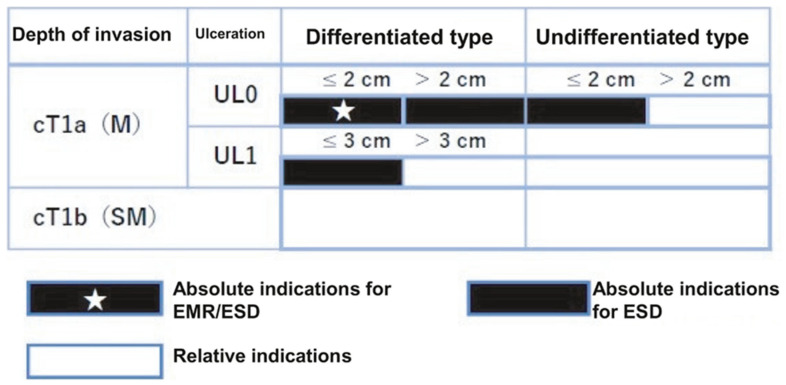
Indications for endoscopic treatment for early gastric cancer (cited from [15]), cT1a (M), intramucosal cancer (preoperative diagnosis), cT1b (SM), submucosally invasive cancer (preoperative diagnosis). UL, finding of ulceration (or ulcer scar); UL0, absence of ulceration or ulcer scar; UL1, presence of ulceration or ulcer scar.

In addition, if the lesion deviates from the “absolute indication” or “expanded indication” lesion, endoscopic resection should be performed as a “relative indication” lesion only if informed consent is obtained after sufficient explanation that the standard treatment is surgical gastrectomy and that the risk of lymph node metastasis remains (Figure 3). Therefore, to determine the treatment strategy, it is necessary to diagnose the histological type, size, depth of invasion, and presence or absence of ulceration or ulcer scar.

The histological type of cancer (differentiated carcinoma vs. undifferentiated carcinoma) is diagnosed via biopsy as described above; however, the histological type can be predicted endoscopically. In general, elevated and protruded lesions are the differentiated type; among flat and depressed lesions, redness tones often indicate the differentiated type, and white tones indicate the undifferentiated type. It has been reported that differentiated (fine network pattern) and undifferentiated types (corkscrew pattern) can be estimated to some extent using band-limited light magnification observation [16]. When endocytoscopy was applied in comparison with magnifying endoscopy in NBI, the accuracy of histological diagnosis increased from 72.2% to 78.8% [17].

It has been pointed out that there is an error in the size measured when using the endoscopic observation method. Therefore, on the premise that the size will be determined after the histological findings of the resected specimen are finally known, the size is diagnosed with reference to the biopsy forceps diameter (usually 2.5 mm in the closed state and 8 mm in the open state). Ulcerative findings are diagnosed via the observation of obvious active ulcers or ulcer scars in the lesion. An ulcer is a defect of the mucosal layer or deeper, and an active ulcer refers to an open ulcer with a certain depth of white moss, excluding shallow erosion. In addition, ulcers in the healing or scar phase are also defined as having ulcerative findings when there is obvious mucosal fold convergence at one point.

Although endoscopic ultrasound is useful as a means of auxiliary depth diagnosis for early gastric cancer, the additional effect is low. It is reported that accuracies of conventional endoscopy and a miniature ultrasound probe (12 MHz) for submucosal invasive carcinoma (T1b) were 79.6% and 82.0%, respectively (*p* = 0.108) [18]. Moreover, from the point of view of labor and medical cost, endoscopic ultrasound is rarely performed in actual clinical practice; auxiliary depth is usually estimated using white light observation. In the case of elevated and protruded lesions, if they exceed 2 cm, there is a possibility of T1b, but if the surface is smooth and regular, and erosion and redness are not observed, it is likely to be T1a regardless of size. In the case of flat and depressed lesions, a deep depression, hardness, unstructured surface, nodules of unequal size in the depression, and significant redness are findings indicative of T1b.

When endoscopic resection is planned, it is necessary to accurately diagnose the extent of horizontal cancer to determine the resection area. In this case, chromoendoscopy with indigo carmine is basically performed, but in recent years, it has been reported that better diagnostic accuracy can be obtained via band-limited light magnification observation (89.4% vs. 75.9%, *p* = 0.0071) [19]. The diagnosis of the extent of horizontal cancer, especially in relation to some moderately to poorly differentiated carcinomas and signet ring cell carcinoma, may be difficult only via endoscopic observation, and it is desirable to take a biopsy from the unclear boundaries to confirm cancer margins.

## 5. Types of Endoscopic Resection for Early Gastric Cancer

**Figure 4:**
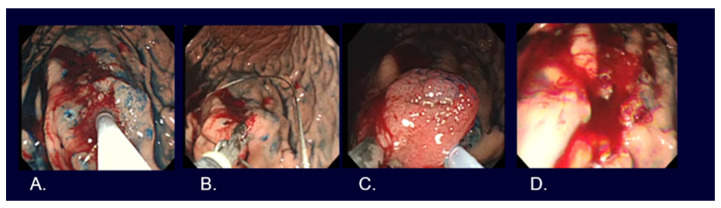
Strip biopsy method for early gastric cancer. (**A**) Submucosal injection after marking around the lesion located in the greater curvature of the upper gastric body. (**B**) Grasping the lesion with grasping forceps in the left working channel and a semi-lucent snare in the right working channel by using a double channel endoscope. (**C**) Pulling the grasping forceps while adjusting the lesion entrapped in the snare. (**D**) Mucosal defect after resection.

**Figure 5:**
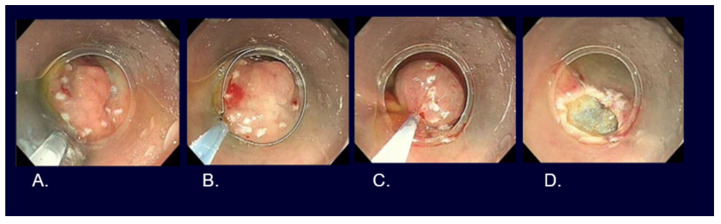
Cap-EMR method for early gastric cancer. (**A**) Submucosal injection after marking around the lesion located in the greater curvature of the lower gastric body. (**B**) Pre-looping a semi-lucent snare to the distal rim of EMR cap. (**C**) Entrapping the lesion after sucking the lesion in the EMR cap. (**D**) Mucosal defect after resection.

**Figure 6:**
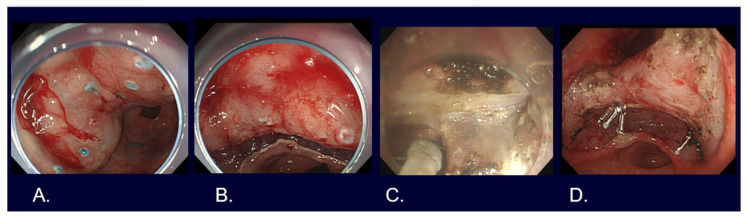
ESD method for early gastric cancer. (**A**) Submucosal injection after marking around the lesion located in the lessor curvature of the gastric angle. (**B**) Mucosal incision around the lesion outside the markings. (**C**) Submucosal dissection beneath the lesion. (**D**) Mucosal defect after resection.

EMR, such as strip biopsy [20] and Cap-EMR [21], and ESD [2] are the main resection methods used for early-stage gastric cancers (Figure 4, Figure 5 and Figure 6).

Although no studies have examined whether treatment outcomes differ between EMR and ESD in randomized controlled trials, meta-analyses, mainly in retrospective studies, have shown that ESD generally provides a better en bloc resection rate (odds ratio of 9.69) and non-local recurrence rate (odds ratio 0.10) than EMR [22]. According to the Treatment Guidelines, either EMR or ESD is acceptable for lesions that are determined to be ≤2 cm, cT1a, and differentiated carcinoma with UL0; however, it has been reported that the rate of en bloc resection in EMR (63.6%) is significantly lower than that of ESD (91.3%) when the tumor size exceeds 1 cm [23].

Multi-piece resection not only increases the rate of local recurrence but may also prevent an adequate pathologic evaluation of resected specimens; thus, ESD is currently the choice for most lesions. In addition, though various electrocautery devices for ESD have been developed, all of them have advantages and disadvantages; therefore, at present, it is important to use the most suitable electrocautery devices as required under relevant clinical circumstances according to the preference of the endoscopists and the location and/or nature of the lesion for safe and reliable ESD.

## 6. Evaluation of Curability after Endoscopic Resection

**Figure 7:**
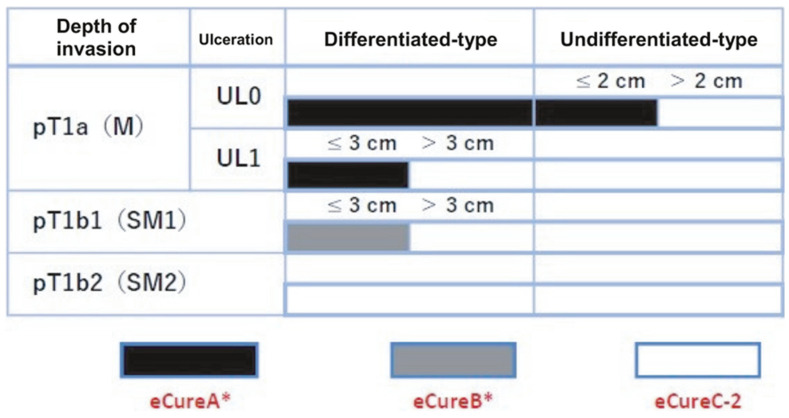
Evaluation of curability for early gastric cancer (cited from [15]). * Confined to en bloc resection and HM0, VM0, Ly0, and V0. pT1a (M), intramucosal cancer (histopathological diagnosis); pT1b (SM), submucosally invasive cancer (histopathological diagnosis). UL, finding of ulceration (or ulcer scar); UL0, absence of ulceration or ulcer scar; UL1, presence of ulceration or ulcer scar.

The curability of EMR and ESD is determined by two factors: the completeness of local cancer excision and the likelihood of lymph node metastasis (Figure 7).

When the lesion is resected en bloc, the following conditions need to be fulfilled in order for the resection to be classified as endoscopic curability A (eCuraA): (i) predominantly of the differentiated type, pT1a, UL0, a horizontal stump negative for cancer (HM0), a vertical stump negative for cancer (VM0), no lymphatic vessel infiltration (Ly0), and no venous infiltration (V0), regardless of size; (ii) long diameter ≤ 2 cm, predominantly of the undifferentiated type, pT1a, UL0, HM0, VM0, Ly0, and V0; or (iii) long diameter ≤ 3 cm, predominantly of the differentiated type, pT1a, UL1, HM0, VM0, Ly0, and V0.

When the lesion is resected en bloc and the following conditions are fulfilled, such resection is classified as endoscopic curability B (eCuraB): ≤3 cm in long diameter, predominantly of the differentiated type, pT1b1(SM1) (within <500 μm from the muscularis mucosae), HM0, VM0, Ly0, and V0.

When a lesion meets neither of the above-mentioned sets of conditions for eCuraA or B, it is classified as endoscopic curability C (eCuraC), and is likely to be a remnant tumor. When eCuraC lesions are differentiated-type lesions and fulfill other criteria allowing for their classification into either eCuraA or eCuraB but are either not resected en bloc or have positive HM, they are classified as eCuraC-1. All other eCuraC lesions are classified as eCuraC-2.

The determination of curability is directly related to the subsequent patient management policy. In the case of eCuraA, follow-up is performed using endoscopy once or twice a year; in the case of eCuraB, in addition to endoscopy once or twice a year, abdominal ultrasonography and CT examination are used to check for metastasis.

In the case of eCuraC-1, as the risk of metastasis is low, appropriate methods, such as re-ESD, additional surgical resection, careful follow-up in anticipation of the burn effect at the time of resection, and cauterization (laser, argon plasma coagulation, etc.) are selected, according to the policy of the institution, after sufficient informed consent has been obtained from the patient.

In the case of eCuraC-2, in principle, additional surgical resection is performed. If surgical gastrectomy is not selected for some reason, such as age or comorbidity, it is necessary to obtain the patient’s full understanding and consent after explaining the risk of lymph node metastasis, the possibility of local recurrence or distant metastasis, and the difficulty of curing and poor prognosis in the event of recurrence.

When following up, it is recommended to test for *H. pylori* infection in patients with an unknown status of *H. pylori* infection and then to eradicate *H. pylori* in *H. pylori* positive patients. However, it is necessary to pay attention to the occurrence of metachronous multiple gastric cancer, even after *H. pylori* eradication, although the individuals with eradication of H pylori infection had a lower incidence of gastric cancer than those who did not receive eradication therapy (pooled incidence rate ratio, 0.53; 95%CI, 0.44–0.64). [24]. At present, there is no evidence suggesting that the surveillance method should be changed after endoscopic treatment due to differences in *H. pylori* infection status.

## 7. Endoscopic Resection Results for Early-Stage Gastric Cancer

Based on a Japanese case series of 10,821 lesions in 9616 patients, ESD, EMR, and a hot biopsy were performed in 10,756 lesions (99.4%), 64 lesions (0.6%), and 1 lesion (0.01%), respectively. The median procedure time with interquartile range was 76 (49–120) minutes, and en bloc and R0 resections were obtained in 10,739 lesions (99.2%) and 9914 lesions (91.6%), respectively. In terms of complications, postoperative bleeding, intraoperative perforation, and delayed perforation were encountered in 426 lesions (4.4%), 218 lesions (2.3%), and 40 lesions (0.4%), respectively. Blood transfusion and emergency surgery were necessary in 69 lesions (0.7%) and 23 lesions (0.2%), respectively [25].

If perforation occurs during endoscopic resection, endoscopic clip closure should be tried first. If it is successful, successive conservative managements such as the administration of antibiotics with abstinence from eating and drinking for a few days is permittable [26]. However, if the perforation cannot be closed, or if peritonitis is suspected even if it can be closed, it is necessary to consult a surgeon and consider surgical indications.

Intraoperative bleeding, including minor cases, is almost inevitable in ESD. Since there is no established definition of the bleeding, it should be noted that there are slight differences in the definition of bleeding rate described above by researchers; some researchers may count the cases when, for example, hemoglobin drops by ≥2 g/dl before and after endoscopic resection, blood transfusion is required, the vomiting of blood is observed after endoscopic resection, and/or gastric blood retention or bleeding from a postoperative ulcer is observed during emergency endoscopy. For bleeding during ESD, coagulation hemostasis with hemostatic forceps, which does not prevent the continuation of resection after hemostasis, is desirable, but depending on the situation, the clip method and local injection method are also options. By combining nine variables (4 points for warfarin and direct oral anticoagulants; 3 points for chronic kidney disease with hemodialysis; 2 points each for P2Y12 receptor antagonist and aspirin; 1 point each for cilostazol, a tumor size > 30 mm, lower third in tumor location and presence of multiple tumors; and −1 point for interruption of each kind of antithrombotic agent), rates of post-ESD bleeding could be well predicted at low risk (2.8%, 0 to 1 points), intermediate risk (6.1%, 2 points), high risk (11.4%, 3 to 4 points) and very high risk (29.7%, ≥5 points), respectively [27]. In order to prevent postoperative bleeding, proton pump inhibitors, H2 blockers, or potassium-competitive acid blockers are administered, but the preventive effect is not sufficient. In recent years, new attempts have been made, such as covering the mucosal defect with a PGA sheet and fibrin glue [28] and stitching the mucosal defect with an indwelling snare and clip [29].

Recently, the long-term outcomes of the Japanese case series described above have been elucidated. The overall 5-year OS was 89.0% (95% CI, 88.3–89.6%). A multivariate analysis revealed no significant differences in the hazard ratio of the mortality of eCuraA subgroup A2 ((i) with >2 cm and eCuraA (iii)) (1.03 [95% CI, 0.87–1.21]), eCuraA subgroup A3 (ii)(1.18 [95% CI, 0.68–2.07]), and eCuraB (1.09 [95% CI, 0.80–1.49]) compared with that of eCuraA subgroup A1 ((i) with ≤2 cm). However, the hazard ratio of the mortality of eCuraC (1.41 [95% CI, 1.21–1.65]) was significantly higher than that of eCuraA subgroup A1 ((i) with ≤2 cm) [30].

## 8. Future Perspectives on Endoscopic Diagnosis and Treatment for Early Gastric Cancer

In 2014, the Cancer Genome Atlas (TCGA) network reported that gastric cancer can be classified into four different molecular subtypes [31]. This was the first step in classifying gastric cancer based on specific genomic abnormalities rather than considering it as a single disease, indicating the possibility of developing personalized medicine.

Under the said classification, EB virus-positive gastric cancer was classified as a subtype that accounted for 9% of the total, along with microsatellite instability gastric cancer, genomic stability gastric cancer, and chromosomal instability gastric cancer. The most distinctive feature of EB virus-positive gastric cancer is the suppression of gene expression by the hypermethylation of extensive genomic DNA. Although EB virus-positive gastric cancer has been known to have a good prognosis clinically, recent studies have suggested that EB virus-positive gastric cancer has a low risk of lymph node metastasis, even with T1b, and, thus, it may be cured by local resection without lymph node dissection, provided that there is no vascular invasion [32].

**Figure 8:**
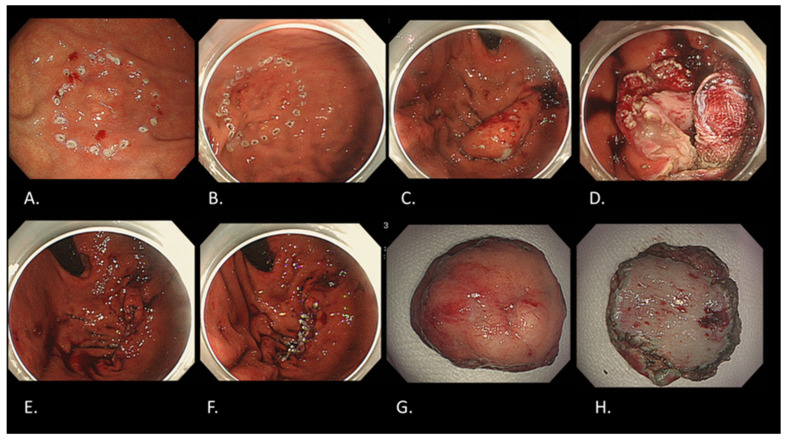
NEWS for a gastric cancer with fundic gland type. (**A**) Marking around the lesion located in gastric fundus on the day before NEWS by gastroscopy. (**B**) Additional markings around the previous markings during NEWS by gastroscopy. (**C**) Sero-muscular incision with complete defect closure by laparoscopy. (**D**) Muco-submucosal incision by gastroscopy-like ESD. (**E**) Complete removal with full-thickness resection by gastroscopy. (**F**) Complete mucosal closure with endoscopic clipping by gastroscopy. (**G**) Resected specimen observed from mucosal side. (**H**) Resected specimen observed from serosal side. Final histological diagnosis is adenocarcinoma (tub2 > tub1), Type 0-IIc, 15 × 10 mm, pT1b2 (540 um), UL(−), ly0, v0.

In 2010, the concept of gastric cancers with the funding gland type was newly proposed, the category of which in the TGCA classification has not been elucidated so far. Gastric cancers of this type have a good prognosis, suggesting that T1b, like EB virus-positive gastric cancer, may be cured by local resection without lymph node dissection [33]. In some gastric cancers, it is possible to evaluate curability differently from the uniform curability standard stipulated in the Treatment Guidelines. In the future, ESD or technology that employs ESD may be widely applied to deep submucosal invasive carcinoma. This is expected to establish technology that can reliably perform the batch excision of deep submucosal infiltrating cancers with a negative vertical stump and technology that enables endoscopic full-thickness resection of the gastric wall, as well as the development of endoscopic equipment that enables said resection. Currently, prior to such breakthroughs, as a new method for the full-thickness resection of the stomach wall, the concept of laparoscopic and endoscopic cooperative surgery (LECS) for gastric submucosal tumors (mainly gastrointestinal stromal tumors (GISTs)), in which an endoscope is used in combination with laparoscopic surgery, has been established and widely practiced [34]. As one of the LECS procedures, non-exposed endoscopic wall inversion surgery (NEWS) was developed (Figure 8) [35]. Compared to other LECS procedures, NEWS has two distinct characteristics: it is possible to set the resection range more accurately, and there is no need to worry about intra-abdominal infection or tumor seeding, as there is no open connection between the stomach and the abdominal cavity. The future development of NEWS is greatly anticipated [36]. There is accumulating evidence of artificial intelligence (AI) for detection, differential diagnosis, and prediction of the demarcation lines and depth of invasion of EGCs [37]. Although recent developments in AI in EGC are still image-based, the future direction must be not only assistance of endoscopic diagnosis but also treatment navigations, prediction of risks of gastric cancer developments, and so on, with combinational use of other available data such as clinical, histological, epigenetic and genetic data.

## 9. Conclusions

In this review, the current status and future prospects of endoscopic diagnosis and treatment for gastric cancer were outlined pursuant to the latest guidelines. In combination with the knowledge of a high-risk group, the diagnostic yields of gastric cancer have significantly improved by magnifying endoscopic observation using band-limited light. ESD enables us to resect the majority of EGCs without lymph node metastasis in an en bloc fashion with acceptable procedural risks. We are entering an era in which tailor-made medical care can be provided in the field of endoscopy according to the patient’s situation, as can be seen from the achievements of endoscopic equipment and technology, as well as the deepening of knowledge about gastric cancer.
